# *In Vitro* and *In Vivo* Antimalarial Activity Assays of Seeds from *Balanites aegyptiaca*: Compounds of the Extract Show Growth Inhibition and Activity against Plasmodial Aminopeptidase

**DOI:** 10.1155/2011/368692

**Published:** 2011-05-25

**Authors:** Peter Kusch, Susanne Deininger, Sabine Specht, Rudeka Maniako, Stefanie Haubrich, Tanja Pommerening, Paul Kong Thoo Lin, Achim Hoerauf, Annette Kaiser

**Affiliations:** ^1^Department of Applied Natural Sciences, Bonn-Rhein-Sieg University of Applied Sciences, Justus Von Liebig Street 20, 53359 Rheinbach, Germany; ^2^Institut für Medizinische Mikrobiologie, Immunologie und Parasitologie, Rheinische Friedrich-Wilhelms-Universität Bonn, Sigmund-Freud-Straße 25, 53105 Bonn, Germany; ^3^Institut für Ägyptologie und Altorientalistik, Johannes Gutenberg Universität Mainz, Johann-Friedrich-von-Pfeiffer-Weg 5, 55128 Mainz, Germany; ^4^School of Pharmacy and Life Sciences, Robert Gordon University, St. Andrew Street, Aberdeen AB25 1HG, UK; ^5^Fakultät für Angewandte Naturwissenschaften, University of Applied Sciences, Betzdorfer Str. 2, 50679 Cologne, Germany

## Abstract

*Balanites aegyptiaca* (*Balanitaceae*) is a widely grown desert plant with multiuse potential. In the present paper, a crude extract from *B. aegyptiaca* seeds equivalent to a ratio of 1 : 2000 seeds to the extract was screened for antiplasmodial activity. The determined IC_50_ value for the chloroquine-susceptible *Plasmodium falciparum* NF54 strain was 68.26 *μ*g/*μ*L ± 3.5. Analysis of the extract by gas chromatography-mass spectrometry detected 6-phenyl-2(H)-1,2,4-triazin-5-one oxime, an inhibitor of the parasitic M18 Aspartyl Aminopeptidase as one of the compounds which is responsible for the *in vitro* antiplasmodial activity. The crude plant extract had a *K*_*i*_ of 2.35 *μ*g/*μ*L and showed a dose-dependent response. After depletion of the compound, a significantly lower inhibition was determined with a *K*_*i*_ of 4.8 *μ*g/*μ*L. Moreover, two phenolic compounds, that is, 2,6-di-*tert*-butyl-phenol and 2,4-di-*tert*-butyl-phenol, with determined IC_50_ values of 50.29 *μ*M ± 3 and 47.82 *μ*M ± 2.5, respectively, were detected. These compounds may contribute to the *in vitro* antimalarial activity due to their antioxidative properties. In an *in vivo* experiment, treatment of BALB/c mice with the aqueous *Balanite* extract did not lead to eradication of the parasites, although a reduced parasitemia at day 12 p.i. was observed.

## 1. Introduction

Traditional medicine is still the first point of healthcare for many people in sub-Saharan Africa, where there has been a long and rich tradition of obtaining treatments from herbs and trees. In the case of malaria, Africa's traditional healers use hundreds of indigenous plants for remedies.

Until the 1950s, when synthetic chemistry began to dominate drug research and development (R and D) efforts, most drugs developed and registered in the pharmacopoeia were in fact based on natural products. Plant alkaloids, quinine among them, were the first components of natural herbal remedies to be extracted and refined for more effective use in the early 19th century. Some 150 years later, quinine is still used as front-line therapy for severe malaria, even if it is not the recommended drug for this use when artemisinin combination therapies (ACTs) are available. In this context, it seems to be quite surprising that no African lead has emerged so far. Meanwhile, there are efforts to assess plant remedies against malaria for their application in health care systems [1].

*B. aegyptiaca* (L.) (*Balanitaceae*) is a woody tree growing in various ecological conditions (from 100 mm to 1000 mm annual rainfall), but mainly distributed in semiarid and arid zones in tropical Africa [2]. This tree reaches 10 m (33 ft) in height with a generally narrow form and thorny branches. The tree produces several forms of inflorescence bearing yellow-green bisexual flowers which exude nectar. In Egypt, it is confined to water-receiving sites, that is, wadis and river banks. 

*B. aegyptiaca* has been applied for a variety of indications in traditional medicine. Its fruit has been the basis for an active trade in many centuries because of its oil, which consists of linear and branched chain alkanes while the kernel contains terpenes and sterols (diosgenin), which showed a reduction of liver cholesterol [3].

The bark was formerly used as a fish poison, presumably because of its steroidal saponin content as the active component [4]. Aqueous extracts of the bark showed antibacterial, antifungal and antiviral activity [5], but most notable was the antifungal effect of *B. aegyptiaca* against *Candida albicans* infections [5].

Balanitin-6 and balanitin-7 are diosgenyl saponins from *B. aegyptiaca* kernels and they demonstrated appreciable anticancer effects in human cancer cell lines *in vitro* [6] with a higher antiproliferative potential than etoposide and oxiplatin. The anticancer activity mainly results from a depletion of ATP leading in turn to a major disorganization of actin cytoskeleton [6].

The structures of balanitins, that is, balanitin-1, -2, -3, are composed of steroid glycosides proven to be potent molluscicides for the control of freshwater snails that are carriers of bilharzia [7]. Moreover, the efficacy of *B. aegyptiaca* fruit mesocarp was compared with praziquantel in infected mice with a Sudanese strain of *Schistosoma mansoni* [8]. A single dose of 200 mg/kg body weight reduced the recovery of adult worms significantly. Recently, root-derived callus from *B. aegyptiaca* with 500 ppm extracted saponins showed a larvicidal effect of 100% against *Aedes aegypti* mosquito larvae [9]. 

Over recent years, resistance of malaria parasites to therapy has increased the need to develop alternative treatments. Since *B. aegyptiaca* bark shows promising anti-infective properties, we investigated its potential antiplasmodial activity in seeds and related this activity to its constituents *in vitro*. Although phytochemical analyses and medicinal evaluations have been recently performed for fixed oil from *B. aegyptiaca* fruits [10], *in vivo* biological assays are missing. Here, we monitored parasitemia after application of an aqueous extract obtained from the seeds of the plant *in vitro* and *in vivo* in BALB/c mice.

## 2. Material and Methods

### 2.1. Plant Material

Seeds of *B. aegyptiaca *(*Balanitaceae*) were obtained from Harraz pharmacy in Cairo, collected in the area of Ras Mohammed National Park and botanically identified by Professor Görtz, Biological Institute, University of Stuttgart, Germany.

### 2.2. Soxhlet Extraction

*B. aegyptiaca* seeds (5 g) were powdered and incubated at 37°C in 100 mL distilled water. To facilitate dissolution of the steroid aglyca, squalane solution (1 mL) (Sigma-Aldrich, Munich, Germany) made with 2 N HCl was added, and the mixture was refluxed with 200 mL methanol for 24 hours in a Soxhlet apparatus. The extract was dried with calcium sulfate (CaSO_4_·2H_2_O) (Carl Roth, Karlsruhe, Germany) and filtered. Subsequently, methanol was evaporated by using the rotary evaporator and a vacuum pump. The residue obtained was diluted in 50 mL methanol by sonication and analyzed by gas chromatography-mass spectrometry (GC/MS). 

For the preparation of an aqueous extract, 5 g of powdered *B. aegyptiaca* seeds were incubated at 37°C in 100 mL distilled, autoclaved water for 24 hours in a sealed, sterilized Falcon tube and filtrated in a laminar air flow under aseptic conditions.

### 2.3. GC/MS Instrumentation and Analytical Conditions

GC/MS measurements were made using a 7890A gas chromatograph with a series 7683B autosampler and a series 5975C quadrupole mass spectrometer (Agilent Technologies Inc., Santa Clara, CA, USA) operated in electron impact ionization (EI) mode. The fused silica capillary column, 30 m long, 0.25 mm I. D. with *HP-5MS *stationary phase, film thickness 0.25 *μ*m was used. GC/MS data were processed with the *ChemStation* software (Agilent Technologies) and the *NIST 05* mass spectra library (Agilent Technologies). The temperature of the column was programmed from 80°C (1 min hold) at 10°C min^−1^ to 280°C (50 min hold). Helium 5.0 grade (Westfalen AG, Münster, Germany) was used as a carrier gas. Constant flow of helium of 1.0 cm³ min^−1^ was used during the entire analysis. The temperature of the split/splitless injector was 250°C, and the split flow was 10 cm³ min^−1^. The transfer line temperature was 280°C. The EI ion source temperature was kept at 230°C. The ionization occurred with a kinetic energy of the impacting electrons of 70 eV (electron volts). The quadrupole temperature was 150°C. Mass spectra and reconstructed chromatograms (total ion current [TIC]) were obtained after eluting of solvent (7 min solvent delay) by automatic scanning in the mass range *m*/*z* 30–750 u.

### 2.4. Cultures of *P. falciparum* Isolate NF54

*P. falciparum* isolate NF54, which was obtained from Malaria Research and Reference Center, Manassas, VA, USA, was maintained in continuous culture with gentamicin (40 *μ*g/mL) in petri dishes (5 cm in diameter) with a gaseous phase of 90% N_2_, 5% O_2_, and 5% CO_2_, according to a protocol from Molony et al. 1990 [11] and Trager and Williams 1992 [12]. Parasites were cultured in human erythrocytes (blood group A^+^) in RPMI 1640 medium (Sigma) supplemented with 25 mM HEPES, 20 mM sodium bicarbonate, and 10% heat-inactivated human A^+^ plasma at 10% (v/v) hematocrit. The parasitemia of the infected erythrocytes was determined in Giemsa-stained smears by light microscopy. Parasitemias and morphological forms detected in the cultures were scored visually with a 100-fold oil-immersion objective, counting at least 1,000 erythrocytes to determine the percentage of the infected erythrocytes [13]. A drug-free control (methanol/water 50 : 50% v,v) was used in all experiments.

### 2.5. Monitoring Multiplication and Growth of Plasmodia

The culture was adjusted to a parasitemia of 1.5%. Aliquots (200 *μ*L) were suspended in 2 mL RPMI-medium (Sigma-Aldrich) supplemented with 25 mM HEPES, 20 mM sodium bicarbonate, and 10% heat-inactivated human A^+^ plasma at 10% (v/v) hematocrit, dispensed into 12-well microculture trays, and incubated at 37°C in a gaseous atmosphere of 90% N_2_, 5% O_2_, and 5% CO_2_. Thereafter, growth medium was changed once a day for 4 days, and 2,4-di-*tert*-butyl-phenol and 2,6-di-*tert*-butyl-phenol stock solutions were dissolved in water/methanol mixture (50/50%, v/v) before being diluted in RPMI growth medium in the concentrations given for each experiment. 

Dilution of the crude *Balanite *extract obtained after Soxhlet extraction was performed with RPMI-growth medium as a solvent to protect the RBC membrane from perturbation.

Parasitemia and stage distribution were estimated as triplicates daily from Giemsa-stained smears by counting 1,000 erythrocytes.

### 2.6. Determination of the IC_50_ Values

The 50% inhibitory concentrations (IC_50_s), defined as the drug concentration corresponding to 50% inhibition of parasitic growth were determined by nonlinear regression analysis.

Data obtained from the inhibitor-dependent concentration growth curves were computed into plots with nonlinear regression analysis from *y*-axis (inhibition %) to *x*-axis (inhibitor concentration *μ*M) [14].

### 2.7. Animals

The animal studies were performed according to the guidelines of the German Animal Welfare Act (license number: 50.203. 2-BN). Balb/c2 mice were maintained and bred at the animal facility of the University of Bonn and infected intravenously with 1 × 10^5^ parasitized erythrocytes from a homologous donor mouse, which had been infected with frozen polyclonal stocks of *P. berghei* ANKA (PbA) strain. BALB/c mice developed a Th2 response and did not succumb to cerebral malaria, but died after 10–30 days because of high parasitemia and anemia. 50 *μ*l of the aqueous crude plant extract (1,25 g/100 mL) were applied to the BALB/c mice intraperitoneally (i.p.) on days 3, 2, 1 before infection, at the day of infection, and on days 1, 2, 3, 4, 5, 6, after infection. Control mice were treated with 200 *μ*L PBS-buffer, and the same scheme of administration was applied. 

2,6-di-*tert*-butyl-phenol and 2,4-di-*tert*-butyl-phenol were applied to the BALB/c2 mice intraperitoneally (i. p.) on days 4 and 2 before infection, and on days 2, 4, and 6 before infection. Chloroquine was applied i. p. on days 1–8 post infection in a concentration of 25 mg/kg for each mouse. Giemsa-stained smears were performed on days 1,2, 3,4, 5,7, 6,8, 10, and 12, and 15. 5 mice were treated in each group.

### 2.8. Recloning of *P. falciparum* M18 Aspartyl Aminopeptidase (PFM 18 AAP)

The nucleic acid sequence from *P. falciparum* M18 Aspartyl Aminopeptidase was chemically synthesized by GenScript (NJ, USA) with optimization of codon usage as described by Teuscher et al. (2007) [15]. A subclone was subsequently constructed with BaculoDirect C-terminal linear DNA (Invitrogen, Germany) and transfected into Sf9 (*Spodoptera frugiperda*) cells for protein expression and purification according to Teuscher et al. (2007) [15].

### 2.9. Activity Assay of the *P. falciparum* M18 Aspartyl Aminopeptidase (PFM 18 AAP)

The activity assay was performed according to a high-throughput-screening (HTPS) described by Teuscher et al. 2007 [15] and by the Scripps Research Institute Molecular Screening Center (http://mlpcn.florida.scripps.edu).

Prior to the start of the assay, 2.5 *μ*L of assay buffer (50 mM Tris HCl pH 7.5, 4 mM CoCl_2_, 0.1% BSA) containing 5 *μ*g/mL recombinant PFM 18 AAP were dispensed into a 12-well microtiter plate. Next, 37 *μ*L of the crude *Balanite* extract and the “depleted Balanite extract” where 6-phenyl-2(H)-1,2,4-triazin-5-one oxime was absent were added as a control reaction to the appropriate wells. Depletion of the extract was performed by liquid chromatography-mass spectrometry (LC/MS), and fractions were rechecked by GC/MS as described within for the presence and absence of the 6-Phenyl-2(H)-1,2,4-triazin-5-one oxime.

The plates were incubated for 30 minutes at 25°C. The assay was started by dispensing 2.5 *μ*L of 100 *μ*M H-Glu-NHMec as a fluorogenic peptide (GenScript, NJ, USA) in buffer (50 mM Tris HCl, pH 8.8) into all wells. Fluorescence was read immediately (*T*_0_) on the ViewLux (Perkin-Elmer, MA, USA) and again after 90 minutes (*T*_90_) of incubation at 25°C. Prior to further calculations, *T*_0_ was substracted from *T*_90_. Three different experiments for each concentration were performed.

### 2.10. Statistics

Statistical analyses were performed using GraphPad Prism software to evaluate the *in vivo* inhibitor experiments performed with BALB/c mice. Normality of data distribution was assessed by the Kolmogorov-Smirnov normality test. Nonparametrically distributed data were analyzed using the Mann-Whitney test for differences between the aqueous *Balanite *extract and the chloroquine treated group.

## 3. Results

*Crude extracts from extracted Balanites aegyptiaca seeds show antiplasmodial activity in the chloroquine susceptible P. falciparum strain NF54 in vitro*.

**Figure 1:**
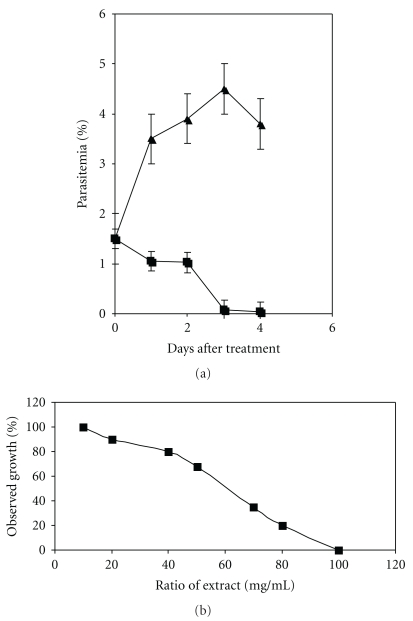
(a) Growth of the chloroquine-susceptible *P. falciparum* strain NF54 in the presence of a diluted extract from *Balanite* seeds obtained from a 1 : 2000 ratio of seeds to extract. Parasites were grown in 12-well microculture trays. Triangles indicate parasitemias of the untreated NF54 control while rectangles indicate the drug-treated strain. Each point represents the mean value of four different determinations with ± standard deviations. (b) Dose-response curve of the crude extract from *Balanite* seeds (extract mg/mL) versus the percentage of the determined parasitemia.

*In vitro* inhibition of growth of the chloroquine susceptible *P. falciparum* strain NF54 was tested with a crude extract from *B. aegyptiaca *(the amount equivalent to 1 : 2000 of a seed) obtained by Soxhlet extraction which led to an eradication of the parasite within 3 days (Figure 1(a)) and a determined IC_50_ of 68.26 *μ*g/*μ*L ± 3,5 (Figure 1(b)). By contrast, the untreated NF54 strain showed normal growth. The crude *Balanite* extract and a dilution of the extract, in which the ratio of seeds to the extract was 1 : 200, lysed red blood cells (RBC; data not shown). Therefore, the amount equivalent to 1 : 2000 of a seed was applied with RPMI-medium as a solvent to avoid any perturbation of the erythrocyte membrane.

**Figure 2:**
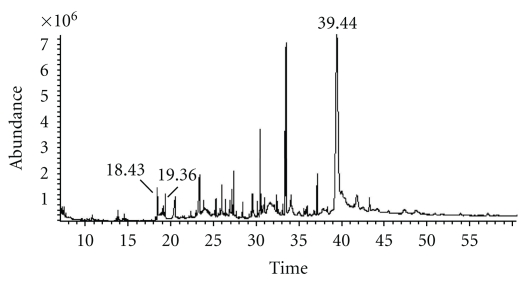
Chromatogram of the total ion current (TIC) obtained by GC/MS analysis of the crude *Balanite* extract. The *x*-axis of each GC/MS chromatogram represents the minutes of retention. The chromatogram shows the detected substances with retention times of 2,6-di-*tert*-butyl-phenol (18.43 min), 2,4-di-*tert*-butyl-phenol (19.36 min), and 6-phenyl-2(H)-1,2,4-triazin-5-one oxime (39.44 min).

**Figure 3:**
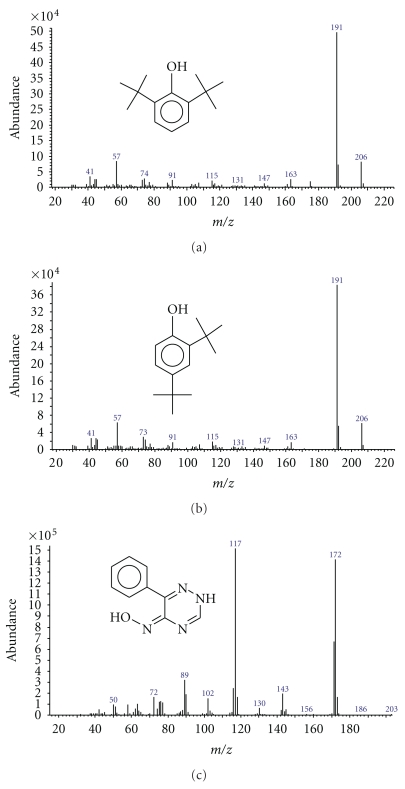
Mass spectra and structural formula of the identified compounds, using pure standard substances and the *NIST 05* mass spectra library. The *x*-axis of each mass spectrum shows the *m*/*z* (mass to charge) units. (a) 2,6-di-*tert*-butyl-phenol, (b) 2,4-di-*tert*-butyl-phenol, and (c) 6-phenyl-2(H)-1,2,4-triazin-5-one oxime.

Figure 2 shows the chromatogram of the total ion current (TIC) obtained by GC/MS analysis of the crude *Balanite* extract to identify and characterize the active compounds of the crude extract after Soxhlet extraction. Figures 3(a)–3(c) represent the mass spectra of the identified peaks using pure standard substances and the peaks of the *NIST 05* mass spectra library of compounds for comparison and to elucidate the chemical structures of the detected substances 2,6-di-*tert*-butyl-phenol (18.43 min), 2,4-di-*tert*-butyl-phenol (19.36 min), and 6-phenyl-2(H)-1,2,4-triazin-5-one oxime (39.44 min), respectively. Ion [M]^+.^ at *m/z* 206, the fragment ion [M –CH_3_]^+^ at *m/z* 191 and the [C_4_H_9_]^+^ ion at *m/z *57 are characteristic for both di-*tert*-butyl-phenol isomers (Figures 3(a)–3(b)). 

**Figure 4:**
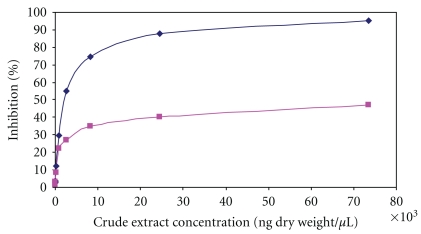
Dose-response curve of crude *Balanite* extract on *P. falciparum* M18 Aspartyl Aminopeptidase (PFM 18 AAP). Different concentrations of the crude *Balanite* extract (*μ*g/mL) were applied in the *in vitro* activity assay of PFM 18 AAP enzyme as described within the experimental section. The percentage of inhibition (*y*-axis) was plotted versus the extract concentration (*x*-axis). The blue curve represents the crude *Balanite* extract with 6-phenyl-2(H)-1,2,4-triazin-5-one oxime while the pink curve shows the depleted extract where the compound is absent.

The crude *Balanite* extract inhibited recombinant M18 Aspartyl Aminopeptidase from *Plasmodium falciparum* (PFM18AAP) with a determined *K*_*i*_ of 2.34 *μ*M. The assay was performed with a commercially available fluorogenic peptide substrate H-Glu-NHMec. Cleavage of the substrate by recombinant PFMAAP enzyme liberates the NHMec from the peptide, leading to increased fluorescence. Different concentrations of the crude plant extract (Figure 4, blue curve) were applied to investigate the dose response of recombinant PFMAAP. Inhibition of PFM18AAP was strictly dose dependent. In parallel, we determined the inhibitory effect of recombinant PFM18AAP after fractionation of the Soxhlet extract pooling fractions in which 6-phenyl-2(H)-1,2,4-triazin-5-one oxime was absent (Figure 4, pink curve). The absence of the compound was confirmed by GC/MS analysis. The pink curve in Figure 4 shows that depletion of 6-phenyl-2(H)-1,2,4-triazin-5-one oxime from the extract leads to a 50% lower inhibition at concentrations ranging from 2.7 ng dry weight per ml to 73.5 ng dry weight per mL compared to the extract where the compound was present. The determined *K*_*i*_ was 4.8 *μ*g/*μ*l.

Derivatives of 6-phenyl-2(H)-1,2,4-triazin-5-one have been recently identified as important inhibitor lead structures for recombinant plasmodial M18 aminopeptidase (PfM18AAP) in a biochemical high-throughput screening assay (HTPS) with fluorogenic peptide substrate (The Scripps Research Institute Molecular Screening Center, http://mlpcn.florida.scripps.edu). The derivatives of 6-phenyl-2(H)-1,2,4-triazin-5-one that is, 3-(benzylamino)-4-ethyl-6-phenyl-1,2,4-triazin-5-one oxime were applied in a concentration of 7.35 *μ*M and inhibited the recombinant enzyme to 31%. This inhibitory effect has been attributed to formation of metal chelates with the cocatalytic zinc metal ions of the parasitic enzyme. Taken together, these results suggest that the detected 6-phenyl-2(H)-1,2,4-triazin-5-one oxime might contribute to the antiplasmodial activity. 

**Figure 5:**
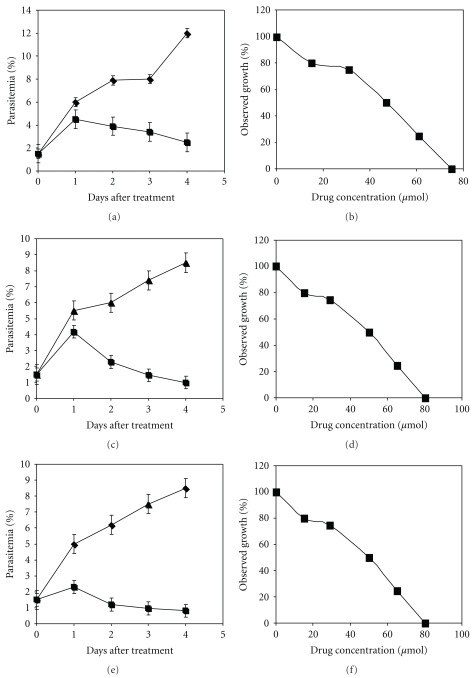
*In vitro* inhibition and dose-response curves of chloroquine-susceptible *P. falciparum* strain NF54. Parasitemias determined after inhibition with (a) 100 *μ*M 2,6-di-*tert*-butyl-phenol, (c) 100 *μ*M 2,4-di-*tert*-butyl-phenol, and (e) a combination of both compounds with 100 *μ*M each. Triangles indicate parasitemias of the untreated NF54 control while rectangles indicate the drug-treated strain. Each point represents the mean value of four different countings with ± standard deviations. Dose-response curves were obtained after inhibition with the compounds in the range of 10 *μ*M–100 *μ*M and IC_50_ values were determined. (b) 2,6-di-*tert*-butyl-phenol, determined IC_50_ = 50.29 *μ*M ± 3, and (d) 2,4-di-*tert*-butyl-phenol, determined IC_50_ = 47.82 *μ*M ± 2,5, and (f) a combination of both compounds determined IC_50_ = 42.5 *μ*M ± 3.

The occurrence of the two phenolic compounds prompted us to perform *in vitro* inhibitor and dose-response experiments with *P. falciparum *strain NF54 at concentrations from 10 mM to 100 mM. For this experiment the commercially available pure phenolic compounds (Fluka, Germany) were applied. Inhibition of growth started at a concentration of 10 mM independently of the compound. The most prominent growth arrest was observed at a concentration of 100 mM with either 2,4-di-*tert*-butyl-phenol or 2,6-di-*tert*-butyl-phenol (Figures 5(a) and 5(c), resp.). The determined IC_50_ values were 50.29 *μ*M ± 3 for 2,6-di-*tert*-butyl-phenol (Figure 5(b)) and 47.82 *μ*M ± 2,5 for 2,4*-*di*-tert-*butyl-phenol (Figure 5(d)), respectively.

A complete eradication of the parasite was not observed, suggesting that these compounds may contribute to the biological activity of the crude plant extract. 2,4-di-*tert*-butyl-phenol was more potent than the 2,6-substituted compound but did not lead to complete eradication of the parasite. In the next set of experiments, both compounds (100 mM each) were applied for combined treatment. The combination of both compounds showed an IC_50_ value of 42.5 *μ*M ± 3 (Figure 5(f)). Taken together, these results suggest that the combination of both drugs appear to have an additive effect. 

*An aqueous extract from B. aegyptiaca seeds and the two phenolic compounds *2,4-di-*tert*-butyl-phenol and 2,6-di-*tert*-butyl-phenol *tested in vivo in BALB/c mice do not reduce parasitemia*. 

**Figure 6:**
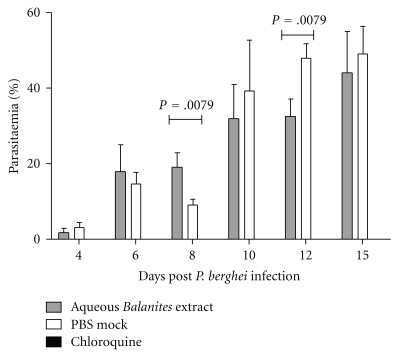
Parasitemia of *Plasmodium berghei*-infected ANKA strain-infected BALB/c mice after treatment with 50 *μ*L of the aqueous *Balanite* extract (1,25 g/100 mL) in comparison to the PBS-buffer-treated control. Administration was performed intraperitoneally on days 3, 2, and 1 before infection, at the day of infection and on days 1, 2, 3, 4, 5, 6, 7, and 8 after infection. Giemsa-stained smears were performed on days 4, 6, 8, 10, 12, and 15.

Hitherto, no *in vivo* biological activity assay has been performed to investigate antiplasmodial activity of the* Balanite *extract. Therefore, we monitored *in vivo* protection in BALB/c infected mice after infection with 10^5^ parasitized erythrocytes of *P. berghei* ANKA (PbA) strain after administration of the *Balanite* extract and the two phenolic compounds, that is, 2,4-di-*tert*-butyl-phenol and 2,6-di-*tert*-butyl-phenol. The crude aqueous plant extract (50 mL of 1,25 g dry extract/100 mL solvent) was intraperitoneally applied on days 3, 2, and 1 before infection, at the day of infection, and on days 1,2, 3,4, 5,6, 7, and 8 after infection. The two phenolic compounds (each dose was 6 mg/kg body weight) were intraperitoneally applied on days 4 and 2 before infection and on days 2, 4, and 6 after infection. At day 8 p.i., parasitemia was significantly increased in mice treated with the extract whereas, at day 12 p.i., a reduction could be observed compared to controls (Figure 6). Loss of body weight is an indicator of malaria infection. In both untreated and extract-treated animals, a reduction of weight could be observed whereas chloroquine treatment prevented the weight loss (data not shown). In a parallel experiment, we monitored the survival rate of BALB/c infected mice with each of the phenolic compounds in a dose of 6 mg/kg body weight (data not shown). However, both phenolics did not reduce parasitemia in comparison to the control and, therefore, did not prolong the survival of *P. berghei* infected mice.

## 4. Discussion

*B. aegyptiaca* (*Balanitaceae*) is a widely distributed plant in Africa and has been reported to show a lot of different biological activities, which were attributed to its saponin constituents [16] mainly deriving from diosgenin as the sole aglycone. Recent investigations proved a medicinal evaluation for the fixed oil of *B. aegyptiaca* fruits *in vitro* [10]. In the past, moderate *in vitro* antiplasmodial activity has been determined in an extract from *B. aegyptiaca* stem bark with an IC_50_ value of 55 *μ*g/mL [17]. No antiplasmodial activity was found in the mesocarp of the fruits from this species. Since it was reported that a biosynthetic pathway to diosgenin exists in germinating seeds of the plant [18], these results challenged us to investigate an extract from *Balanite *seeds obtained by Soxhlet extraction.

An aqueous dilution from the original extract in which the ratio of seeds was equivalent to a ratio of 1 : 2000 seeds was tested for *in vitro* inhibition of the chloroquine-susceptible *P. falciparum* strain NF54, resulting in an IC_50_ value of 68.26 *μ*g/*μ*L ± 3,5. Although no saponin glycosides deriving from the aglycone diosgenin were identified by GC/MS (Kaiser unpublished), the crude, undiluted extract seems to contain constituents which lyse RBCs. 

*In vitro* activity is associated (Figure 4 blue curve) with the occurrence of the oxime of 6-phenyl-2(H)-1,2,4-triazine-5-one, since the absence of the compound in the *Balanite* extract leads to a 50% lower inhibition of recombinant plasmodial M18 Aspartyl Aminopeptidase (PfM18AAP) (Figure 4, pink curve) compared to the crude extract where the oxime of 6-phenyl-2(H)-1,2,4-triazine-5-one was present (Figure 4 blue curve). However, the concentration of the compound in the extract was too low for a successful isolation and a proof of principle assay inhibiting growth of *Plasmodium in vitro*. To our knowledge, there are no previous studies with the compound in the literature so far.

Taken together, these data are supported by the determined *K*_*i*_ of 2.35 *μ*g/*μ*L of the crude *Balanite *extract and the extract which was depleted of the 6-phenyl-2(H)-1,2,4-triazine-5-one oxime with a determined *K*_*i*_ of 4.8 *μ*g/*μ*L. 

Aminopeptidases are metalloproteases which cleave aminoterminal amino acids during protein biosynthesis. The Aspartyl Aminopeptidase exhibits exopeptidase activity exclusively against the N-terminal acidic amino acids glutamate and aspartate from the host hemoglobin-derived peptides. The enzyme is mainly located in the cytosol but can also be trafficked to the parasitophorous vacuole. Antisense-mediated knockdown of PfM18AAP results in a lethal phenotype since the *P. falciparum* protein is expressed in all erythrocytic stages [15].

The two phenolic compounds 2,4-di-*tert*-butyl-phenol and 2,6-di-*tert*-butyl-phenol were identified by GC/MS analysis (Figures 2-3) and contribute modestly to the *in vitro* biological activity (Figure 5). The determined IC_50_ values were 50.29 *μ*M ± 3 for 2,6-di-*tert*-butyl-phenol and 47,82 *μ*M ± 2,5 for 2,4*-*di*-tert-*butyl-phenol, respectively (Figures 5(b) and 5(d)). However, the determined IC_50_ value of 42.5 *μ*M ± 3, when both compounds were combined, appears to have a small additive effect but did not lead to the eradication of *P. falciparum in vitro*.

These compounds have been recently associated with the antioxidant effect of a methanolic extract from the centipede *Scolopendra subspinipes mutilans* (*Scolopendridae*) which is used in traditional Chinese and Korean medicine for the treatment of diseases such as spasm, childhood convulsions, diphtheria and tetanus [19]. Moreover, 2,4-di-*tert*-butyl-phenol exhibited antioxidant activity against LDL-oxidation in a biological *in vitro* activity assay [19]. Consistent with the observation that 2,4-di-*tert*-butyl-phenol showed antioxidant activity, the drug exhibited also radical scavenging activity. Recently, the antioxidant capacity was also proven for the oil present in *Balanites aegyptiaca* fruits *in vitro* [10]. 

The two phenolic compounds 2,4-di-*tert-*butyl-phenol and 2,6-di-*tert*-butyl-phenol have been detected in a variety of plant extracts. They occur as aromatic constituents in volatile oil fractions of five different species of Chinese eaglewood [20] such as *Aquilaria sinensis *(*Thymelaeaceae*), in flowers of *Gynura cusimbua *(*Asteraceae*) [21], in flowers of *Clusia* species (*Guttiferae*) [22], and in leaves of *Pereskia bleo* (*Cactaceae*) [23]. Hitherto, the biogenesis of both phenolic compounds in the plant has not been elucidated. A possible precursor molecule which is a structural analogue of these tertiary phenolics might be a butylated hydroxyanisole deriving from the mevalonate pathway.

Secondly, in case of the extract obtained from *Pereskia bleo,* Ali et al. [24] discussed the occurrence of both constituents as an uptake of the compounds from plastic waste being in the soil. We have no data which support the recent findings that the constituents are either of plant origin or due to uptake of both constituents. The content of total phenols in the methanol extract is 3.5% which is comparable to that of *Pereskia bleo* [23] and might protect the fatty oil in the seeds from oxidation.

Although the fixed oil in *B. aegyptiaca* fruits exhibited anticancer activity in lung, liver, and brain human carcinoma cell lines, no cytotoxicity could be detected for the seed extract (data not shown).

Hitherto, only two *in vivo* experiments in mice have been performed with either the pure constituents of the plant or the crude *Balanite* extract [6, 24]. The steroidal saponins balanitin-6 and balanitin-7 increased the survival rate of mice bearing murine L1210 leukemia grafts to the same extent reported for vincristine [6]. 

A second *in vivo* experiment was performed with a crude Balanite extract to demonstrate moderate antihepatotoxic activity in comparison with the standard hepatoprotective silymarin [24]. The lyophilized extract of *Balanites aegyptiaca *was administered orally for 5 consecutive days in a dose of 1 g/kg. On the last day of treatment, a hepatotoxic oral dose of paracetamol (0.6 g/kg) was given and the activity of diagnostic liver enzymes were determined. *B. aegyptiaca* had only a relatively modest hepatoprotective activity [24]. To analyze parasitemia, BALB/c infected mice were treated with a crude, aqueous *Balanite* extract (50 mL of 1,25 g dry extract/100 mL solvent) injected intraperitoneally on a daily basis, beginning on day 3,2, 1 prior to infection, at the day of infection, and continuing until 8 days p.i. Although a decrease in parasitemia could be observed on day 12 (Figure 6), treatment did not clear the parasites over this time and therefore; did not prolong survival. The plasmodial M18 Aspartyl Aminopeptidase inhibitor is present in the extract, however, its concentration might be too low to eradicate the parasite. Toxicity issues prevented the performance of dose escalation studies. The herbicide metamitron is a 4-amino-3-methyl-6-phenyl-1,2,4,triazin-5-one and, therefore, closely related from its structure to the 6-phenyl-2(H)-1,2,4-triazin-5-one oxime. The LD_50_ for metamitron for a female rat is approximately 1.4, and for a male rat it is 2.9 mg/kg body weight. It is a neurotoxic compound. Given that 6-phenyl-2(H)-1,2,4-triazin-5-one oxime has the same effect, it would have enhanced the neurological symptoms in the infected BALB/c mice independently from the parasitic infection. Since there is a lack of knowledge with respect to the half-life, absorption, distribution, and elimination of the three identified compounds in a mammalian organism, we, therefore, conclude that the crude plant extract in its current form and concentration does not reduce parasitemia *in vivo*.
